# Supramolecular Graphene Quantum Dots/Porphyrin Complex as Fluorescence Probe for Metal Ion Sensing

**DOI:** 10.3390/ijms26157295

**Published:** 2025-07-28

**Authors:** Mariachiara Sarà, Andrea Romeo, Gabriele Lando, Maria Angela Castriciano, Roberto Zagami, Giovanni Neri, Luigi Monsù Scolaro

**Affiliations:** 1 Dipartimento di Scienze Chimiche, Biologiche, Farmaceutiche ed Ambientali, University of Messina, V.le F. Stagno D’Alcontres, 31, 98166 Messina, Italy; mariachiara.sara@studenti.unime.it (M.S.); gabriele.lando@unime.it (G.L.); mcastriciano@unime.it (M.A.C.); roberto.zagami@unime.it (R.Z.); 2 CNR-ISMN Istituto per lo Studio dei Materiali Nanostrutturati c/o Dipartimento di Scienze Chimiche, Biologiche, Farmaceutiche ed Ambientali, University of Messina, V.le F. Stagno D’Alcontres, 31, 98166 Messina, Italy; 3 Department of Engineering, University of Messina, C.da Di Dio, 98166 Messina, Italy; giovanni.neri@unime.it

**Keywords:** porphyrin, graphene quantum dots, fluorescence emission, heavy metal ions, supramolecular system

## Abstract

Graphene quantum dots (GQDs) obtained by microwave-induced pyrolysis of glutamic acid and triethylenetetramine (trien) are fairly stable, emissive, water-soluble, and positively charged nano-systems able to interact with negatively charged *meso*-tetrakis(4-sulfonatophenyl) porphyrin (TPPS_4_). The stoichiometric control during the preparation affords a supramolecular adduct, GQDs@TPPS_4_, that exhibits a double fluorescence emission from both the GQDs and the TPPS_4_ fluorophores. These supramolecular aggregates have an overall negative charge that is responsible for the condensation of cations in the nearby aqueous layer, and a three-fold acceleration of the metalation rates of Cu^2+^ ions has been observed with respect to the parent porphyrin. Addition of various metal ions leads to some changes in the UV/Vis spectra and has a different impact on the fluorescence emission of GQDs and TPPS_4_. The quenching efficiency of the TPPS_4_ emission follows the order Cu^2+^ > Hg^2+^ > Cd^2+^ > Pb^2+^ ~ Zn^2+^ ~ Co^2+^ ~ Ni^2+^ > Mn^2+^ ~ Cr^3+^ >> Mg^2+^ ~ Ca^2+^ ~ Ba^2+^, and it has been related to literature data and to the sitting-atop mechanism that large transition metal ions (e.g., Hg^2+^ and Cd^2+^) exhibit in their interaction with the macrocyclic nitrogen atoms of the porphyrin, inducing distortion and accelerating the insertion of smaller metal ions, such as Zn^2+^. For the most relevant metal ions, emission quenching of the porphyrin evidences a linear behavior in the micromolar range, with the emission of the GQDs being moderately affected through a filter effect. Deliberate pollution of the samples with Zn^2+^ reveals the ability of the GQDs@TPPS_4_ adduct to detect sensitively Cu^2+^, Hg^2+,^ and Cd^2+^ ions.

## 1. Introduction

Metal ions are naturally occurring and play a vital role in life. Some, such as sodium, potassium, magnesium, calcium, iron, copper, and zinc, are essential for the proper functioning of the body. However, other metals, referred to as “heavy” metals (such as cadmium, lead, arsenic, and mercury), can be extremely harmful to health and the environment [1,2]. These heavy metals, once introduced into the food chain, tend to accumulate in living organisms, including humans [3,4,5]. This bioaccumulation can severely damage vital organs such as the liver, kidneys, heart, and nervous system and can lead to long-term health problems such as cancer, neurological disorders, and developmental delays.

Therefore, monitoring and detecting heavy metals in the environment, especially in food, is crucial for safeguarding public health. Traditional methods of detecting these pollutants, such as atomic absorption spectroscopy (AAS) or inductively coupled plasma mass spectrometry (ICP-MS), are highly accurate, but they can be expensive, time-consuming, and require specialized equipment. Instead, fluorescent sensors provide a more accessible and cost-effective alternative technology for this purpose [6,7]. These devices can detect heavy metals in various environments by emitting characteristic light correlated to the presence and concentration of the metal ion. Graphene Quantum Dots (GQDs), a class of carbon-based photoluminescent nanomaterials, represent an exciting and promising area of research in the field of sensors and nanomaterials [8,9,10]. Detection of metal ions by using GQD-based sensors relies on several mechanisms that can either enhance or attenuate the fluorescence intensity depending on the specific interactions between the metal ions and the GQDs [11]. These materials, obtained through the decomposition of organic precursors, exhibit good spectroscopic characteristics, stability over time, and are biocompatible and environmentally friendly [12,13]. Among the various methods for synthesizing these systems, the bottom-up approach from organic compounds through thermal decomposition, microwave pyrolysis, plasma treatment, and hydrothermal oxidation is widely used [14,15]. To date, capping agents bearing carboxyl, hydroxyl or other functional groups play a crucial role in the synthesis of GQDs especially in ensuring more stability, solubility and dispersion in various solvents. Amine functionalization plays also a significant role in altering electronic properties, fluorescent emission spectra, and Stokes shift, all of which are crucial for optimizing the performance of GQD-based sensors [16,17,18,19].

In addition, heteroatom doping during synthesis provides a more efficient and consistent way to tailor the properties of GQDs compared to post-synthetic doping [20,21].

Recently, nitrogen-doped graphene quantum dots (NGQDs) and N,K co-doped graphene quantum dots (N,K-GQDs) showing strong fluorescence intensity and high quantum yield have been used for simultaneous detection of Hg^2+^ and Cu^2+^ in water samples [22].

GQDs with their distinctive properties like large surface area, tunable fluorescence, and strong π-conjugation provide excellent building blocks in supramolecular chemistry. Once GQDs are assembled by non-covalent interactions with other molecular components, they can form highly functional and versatile systems for a variety of applications [23,24,25,26,27]. In this respect, porphyrins, due to their fascinating optical properties, are easily modulated through structural modifications, metal incorporation, and aggregation, representing a strategic molecular tool to be efficiently integrated into GQD-based supramolecular systems to design new multifunctional hybrid materials [28,29,30,31,32,33].

**Scheme 1:**
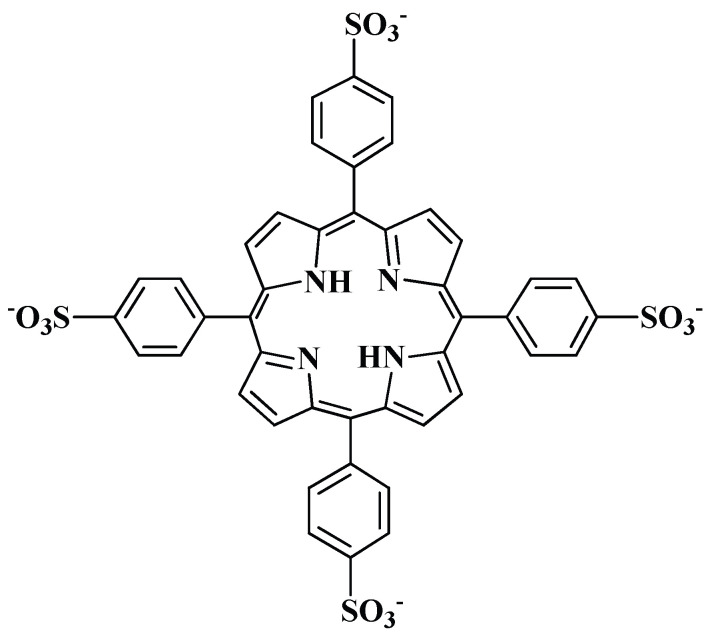
Molecular formula of *meso*-tetrakis(4-sulfonatophenyl) porphyrin (TPPS_4_).

Recent studies on the topic have stimulated a growing interest in the development of such hybrid sensors combining the synergy between these two classes of materials, promising to overcome individual limitations and opening new frontiers in the realization of high-performance devices [34,35,36,37]. The latter exhibits promising applications in photocatalysis [25,34,35], solar energy harvesting [36,37,38], drug delivery [39,40,41,42], medical imaging [43], and selective ion sensing [44,45,46]. Regarding the latter, porphyrins may selectively bind the target metal ion and through synergistic effects, and GQDs can significantly enhance the optical response, allowing for a trace-level ion detection, an approach potentially used in environmental pollution, health diagnostics, food safety, and agriculture. In addition, recent studies have shown that NGQDs can accelerate coordination reactions between metal ions and porphyrins, providing further opportunities for the development of increasingly high-performance sensors. In this respect, Zhang et al. showed an example of NGQDs as a catalyst accelerating the coordination reaction between cadmium (II) and the cationic porphyrin 5,10,15,20-tetrakis(1-methyl-4-pyridinium)porphyrin (TMPyP) to be used as a new optical probe for sensing Cd^2+^ ions [44], while Peng et al. reported an efficient method to catalyze the formation of manganese (II) derivative of TMPyP by using the synergistic effect of trace of NGQDs and Hg(II) ions [47]. Most recently, our research group reported the synthesis of a new type of positively charged GQDs by using the microwave-assisted pyrolysis of glutamic acid and triethylenetetramine tetrahydrochloride (trien) as precursors [48,49]. The nanoparticles easily interact with the water-soluble negatively charged *meso*-tetrakis(4-sulfonatophenyl) porphyrin (TPPS_4_, see Scheme 1), leading to quite stable supramolecular adducts that, as the acidity of the solution increases, allow the diacid species of TPPS_4_ to self-aggregate through the catalytic action of the cationic end groups of nanoparticles as well. TPPS_4_ holds a highly significant and enduring place in the scientific literature across various disciplines for its ability to self-aggregate, particularly into J-aggregates either under controlled environmental factors [50,51,52] or on various nanohybrid platforms [53,54,55], providing customized supramolecular structures with improved or novel properties. Due to the previous results, we now propose to use the supramolecular system (GQDs@TPPS_4_) at neutral pH and in a precise stoichiometric ratio to optimize the emission behavior of both fluorophores (GQDs and porphyrin). The occurrence of GQDs significantly increased the ability to bind a variety of metal ions into the porphyrin core of TPPS_4_. Most specifically, the GQDs@TPPS_4_ system can efficiently sense cupric and mercuric ions by only detecting cadmium (II) and zinc (II) ions at a lower buffer concentration. The presence of adventitious zinc(II) ions in solution can significantly affect chemical reactions and biological processes by complicating the identification of other metal ions in sample [56,57,58]. In this perspective, the effectiveness of the GQDs@TPPS_4_ system was also tested by using both reactants and solvent deliberately polluted with a few tens of micromolar concentration of Zn^2+^ ion. The experimental findings highlight an increased sensitivity of the system in detecting some metal ions, among which Cd(II), Hg(II), and Cu(II) ions emerge.

## 2. Results and Discussions

### 2.1. Sensing of Metal Ions

**Figure 1:**
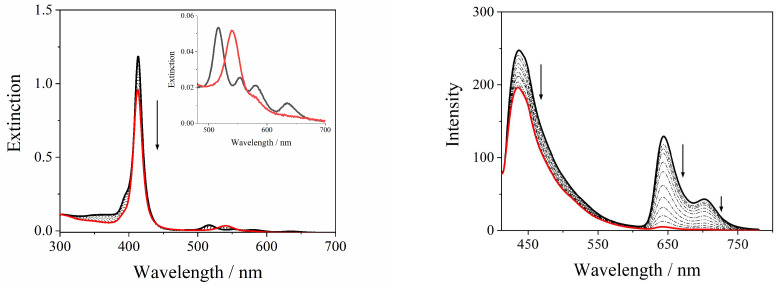
UV/Vis absorbance spectral changes (**left panel**) and corresponding fluorescence emission spectra variation exciting at λ = 380 nm (**right panel**) for GQDs@TPPS_4_ recorded, respectively, at the beginning (solid black line) and at the end (solid red line) of a titration with Cu^2+^ ions carried out at neutral pH (the arrow marks the increasing metal ion concentration). Experimental conditions: [TPPS_4_] = 3 μM; [GQDs] = 0.018 mg/mL; [Cu^2+^] = 0–11 μM; PBS 10 mM, pH = 7; T = 298 K; cell path length 1 cm.

**Figure 2:**
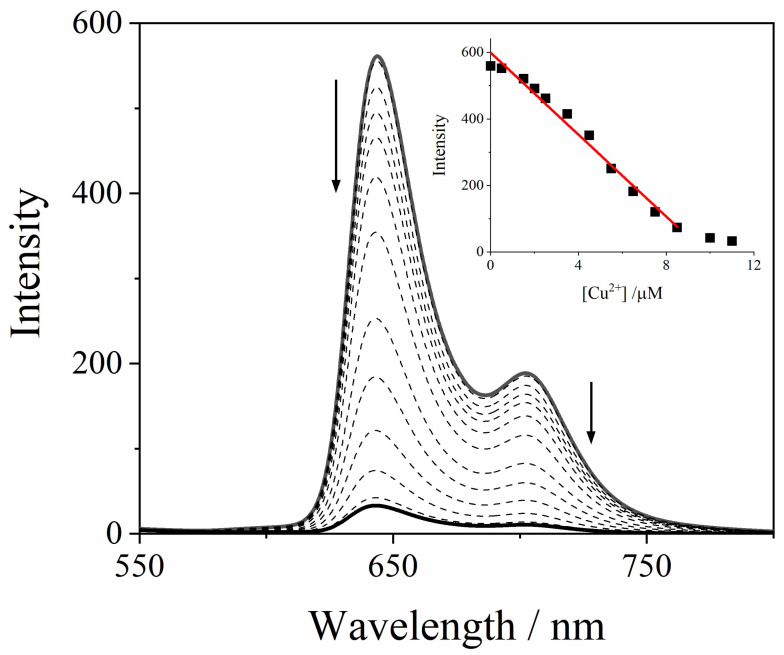
Fluorescence emission spectral variation exciting at λ = 420 nm and corresponding fluorescence intensity changes at 645 nm (**inset panel**) during the titration of GQDs@TPPS_4_ with Cu^2+^ ions at neutral pH, and from which a LoD = 0.059 µM is calculated (the red line represents the linear fit of the data; the arrow marks the increasing metal ion concentration). Experimental conditions: [TPPS_4_] = 3 μM; [GQDs] = 0.018 mg/mL; [Cu^2+^] = 0–11 μM; PBS 10 mM, pH = 7; T = 298 K; cell path length 1 cm.

Previous data have shown, under neutral pH conditions, GQDs employed in this study are positively charged nanometer-sized particles due to the use of trien being a capping agent. Their colloidal stability is proved by the measured zeta-potential (+24 mV) [48]. Under these experimental conditions, GQDs may interact through π-π stacking and electrostatic interaction with aromatic molecules such as negatively charged porphyrins. A titration of the TPPS_4_ with the carbon nanoparticles leads to progressive aggregation of the chromophore and subsequent quenching of its fluorescence emission that could be ascribed to a photo-induced electron transfer from the electron donor excited state of the porphyrin to the acceptor GQDs [48]. In this context, with the aim to test the GQDs@TPPS_4_ system as a potential fluorescent sensor for metal ions, experimental conditions were opportunely chosen to ensure a sufficiently low concentration of nanoparticles so as to preserve the fluorescence emission of the dye by occurrence of self-aggregation. Figure 1 (left panel, line black) shows a typical UV/Vis spectrum of a solution of GQDs@TPPS_4_ (3 μM TPPS_4_ and 0.018 mg/mL GQDs) in 10 mM of PBS at pH 7, which is featured by the presence of a characteristic band at 330 nm attributable to the carbon nanoparticles and a B-band at 414 nm, as well as four weaker Q bands centered, respectively, at 516, 540, 580, and 612 nm, which represent the typical extinction features of TPPS_4_. Figure 1 (right panel, line black) reports the fluorescence emission spectrum corresponding to excitation at 380 nm, whereby it is evident as emission from both GQDs (470 nm) and TPPS_4_ (660 and 710 nm) is simultaneously induced. The stoichiometric ratio used is such that the addition of the anionic porphyrin determines the reversal of the charge of the system, which then becomes globally negative, as substantiated by the data of zeta-potential (−13 mV). Under this changed experimental condition, GQDs@TPPS_4_ may act as a new catalyst aimed to accelerate the coordination reaction between porphyrin and metal ions. In fact, the latter might be found condensed in the close proximity of the semiprotonated primary and secondary amino-groups of trien [59] and, therefore, also nearby the dyes so as to promote the metalation process in close analogy with similar systems that have been reported in the literature [60]. In this perspective, a titration has been conducted by adding micromolar aliquots of Cu^2+^ ions to a solution of GQDs@TPPS_4_, whose final experimental evidence is reported in Figure 1 (left panel, red line). According to the extinction spectra, there are no noticeable changes in the B band, while on the other hand, the pattern of Q bands significantly changes by decreasing the number of bands from 4 to 2 (540 and 580 nm) as the concentration of copper ions increases. Such experimental evidence strongly supports the formation of the metal derivative Cu(II)TPPS_4_ in agreement with the literature data [61]. Fluorescence emission experiments have pointed out such a system can, in principle, behave as a potential sensor of a nonratiometric nature for Cu^2+^ ions. In fact, as shown in Figure 1 (right panel, red line), upon addition of increasing amounts of copper ions, the fluorescence emission intensity of GQDs slightly changes, whereas the corresponding porphyrin emission intensity appears to be completely quenched due to the formation of the copper derivative of the porphyrin on the surface of GQDs. Figure 2 reports the fluorescence emission spectral changes corresponding to excitation at 420 nm and the corresponding progressive quenching of the emission fluorescence intensity (inset) recorded at 645 nm during the titration of the porphyrin with Cu^2+^ in a concentration range from 0 to 11 µM. The detection limit (LoD) was determined from the fluorescence titration data based on a reported method [62]. The good linearity at the micromolar concentration level suggests that the probe GQDs@TPPS_4_ may quantitatively detect small quantities of copper ions with a calculated LoD of about 0.059 µM, a value improved 4-fold compared with TPPS_4_ when alone (LoD = 0.24 µM), as reported in Figure S1.

**Figure 3:**
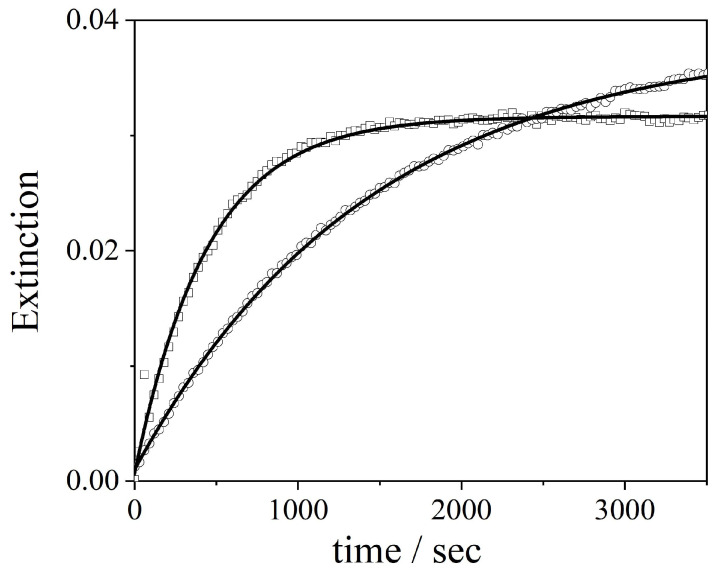
Kinetic traces collected at 540 nm for the metalation of TPPS_4_ porphyrin catalyzed by the presence of GQDs (empty squares) and in bulk (empty circles). Experimental conditions: [TPPS_4_] = 3 μM; [GQDs] = 0.018 mg/mL; [Cu^2+^] = 30 µM; PBS 10 mM, pH = 7; T = 298 K; cell path length 1 cm. The solid lines in the extinction profiles are the best fit of the experimental data according to Equation (1). (GQDs@TPPS_4_): k = (2.25 ± 0.03) × 10^−3^ s^−1^; R^2^ = 0.99. (TPPS_4_): k = (7.0 ± 0.05) × 10^−4^ s^−1^; R^2^ = 0.99.

Additional evidence suggesting GQDs@TPPS_4_ behaves as a catalyst to accelerate the coordination reaction between Cu^2+^ ions and the porphyrin is supported by a comparison of the metalation kinetics performed at fixed copper ion concentration (30 μM), both in the presence and absence of the GQDs (Figure 3). The time evolution of the extinction at 540 nm shows, in both cases, a first-order behavior whose best fit of the experimental data according to Equation (1) allowed us to estimate, in the case of the GQDs@TPPS_4_ system, a rate constant increased by three times with respect to porphyrin in bulk (Figure 3). These results suggest GQDs catalyze the metal ion coordination process.

**Figure 4:**
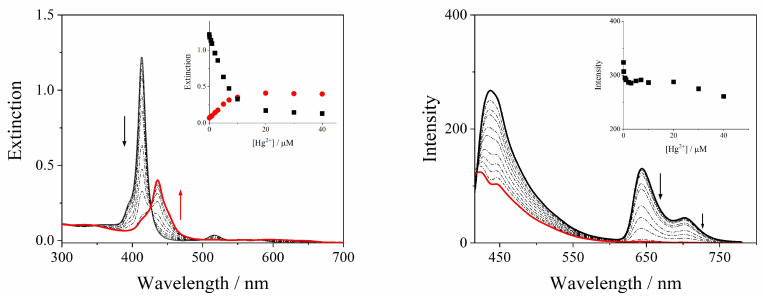
UV/Vis absorbance spectral changes (**left panel**) and corresponding fluorescence emission spectra variation exciting at λ = 380 nm (**right panel**) during the titration of GQDs@TPPS_4_ with Hg^2+^ ions at neutral pH (the arrow marks the increasing metal ion concentration). In the left inset are shown the extinction trends corresponding to 414 nm (black squares) and 436 nm (red circles), whereas the right inset reporting the fluorescence emission evolution at 436 nm properly corrected for the filter effect induced by the absorption of the porphyrin metal derivative. Experimental conditions: [TPPS_4_] = 3 μM; [GQDs] = 0.018 mg/mL; [Hg^2+^] = 0–40 μM; PBS 10 mM, pH = 7; T = 298 K; cell path length 1 cm.

The GQDs@TPPS_4_ system is also able to detect mercuric ions, albeit with a lower sensitivity as compared to the findings with the copper ions. Most specifically, and as readily found in the spectrophometric titration reported in Figure 4 (left panel), the quantitative formation of the metal porphyrin derivative occurs at a concentration of mercuric ions of about 20 μM. The spectroscopic characteristics of Hg(II)TPPS_4_ are in agreement with the literature, displaying a Soret band located at 433 nm and two Q bands, respectively, placed at 590 and 628 nm [63]. The right panel of Figure 4 shows the corresponding fluorescence emission spectra obtained by exciting at 380 nm, by which a marked quenching in the region of porphyrin is evident concomitantly to a noticeable decrease in the fluorescence emission of GQDs. This latter evidence constitutes an artefact arising from the secondary inner filter effect due to the absorption of the major component of porphyrin that results in a loss of emitted fluorescence light. The fluorescence emission data were thus corrected by accounting for the uptake contribution of the porphyrin (see Section 3.2 Methods), and its trend as a function of the increasing concentration of mercuric ions is shown in the inset of Figure 4 (right panel), which highlights the stability of GQD emission in line with that previously observed with cupric ions.

**Figure 5:**
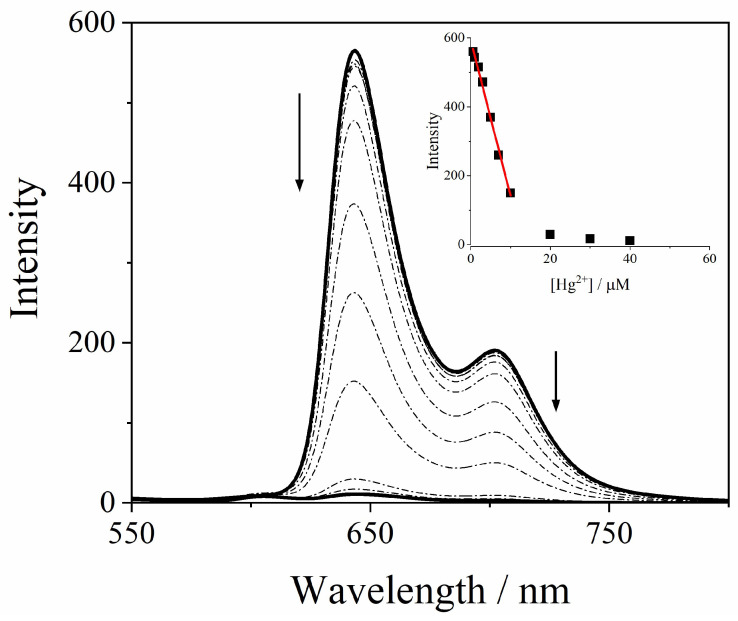
Fluorescence emission spectral variation exciting at λ = 426 nm and corresponding fluorescence intensity changes at 645 nm (**inset panel**) for the titration of GQDs@TPPS_4_ with Hg^2+^ ions at neutral pH, and from which a LoD = 0.078 µM is calculated (the red line represents the linear fit of the data; the arrow marks the increasing metal ion concentration). Experimental conditions: [TPPS_4_] = 3 μM; [GQDs] = 0.018 mg/mL; [Hg^2+^] = 0–40 μM; PBS 10 mM, pH = 7; T = 298 K; cell path length 1 cm.

Figure 5 shows the change in the fluorescence emission spectra by exciting at 426 nm and the corresponding gradual quenching of the fluorescence emission intensity (inset) recorded at 645 nm for the titration of porphyrin with Hg^2+^ ions in the range of concentration from 0 to 40 µM. LoD calculated from fluorescence titration data is 0.078 µM, a value slightly improved with respect to TPPS_4_ alone (LoD = 0.15 µM, Figure S2).

Under the adopted experimental conditions, the GQDs@TPPS_4_ supramolecular system is unable to reveal both the presence of Zn(II) and Cd(II) ions. The reason is attributable to the rather strong interaction that occurs between the phosphate ion used as a buffer that acts as a ligand towards the metal ions, reducing their concentration as free ions. In this regard, an appropriate investigation has been carried out to define the major species distribution that would occur in aqueous solution to evaluate the percentage of the uncomplexed cation available for the interaction with the GQDs@TPPS_4_ system. Data processing of chemical speciation has been performed by using the PyES program (v. 2.0.15) [64]. PyES is a software capable of computing species equilibrium concentration (i.e., free concentration) by solving mass balance equations for all components in solution under the assumption that their total analytical concentrations and equilibrium constants of the chemical species formed in solution are known. Equilibrium concentration of all the species, computed at fixed pH and ionic strength values (pH = 7, I ~ 0.03 M), is provided as pie charts in Figure S3 (analytical concentrations are reported as headers of the chart), whereas equilibrium constants used are provided in Table S1. A careful inspection of the data provided in Table 1 reveals how, in the presence of 10 mM PBS, the percentage of both uncomplexed Cd(II) and Zn(II) ions is very low, thus resulting in the eventual very slow response of the supramolecular system compared to the experimental times adopted in the titration protocol (see methods).

**Figure 6:**
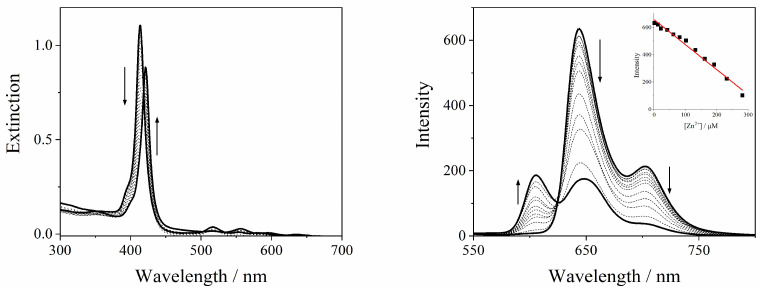
UV/Vis absorbance spectral changes (**left panel**) and corresponding fluorescence emission spectra variation exciting at λ = 418 nm (**right panel**) during the titration of GQDs@TPPS_4_ with Zn^2+^ ions at neutral pH (the arrow marks the increasing metal ion concentration). In the inset of the right panel are reported the corresponding fluorescence intensity changes at 645 nm, from which a LoD = 1.87 µM is calculated (the red line represents the linear fit of the data). Experimental conditions: [TPPS_4_] = 3 μM; [GQDs] = 0.018 mg/mL; [Zn^2+^] = 0–280 μM; PBS 1 mM, pH = 7; T = 298 K; cell path length 1 cm.

Lowering the concentration of PBS from 10 to 1 mM results in a tangible responsiveness of the GQDs@TPPS_4_ system to the presence of both zinc(II) and cadmium(II) ions in virtue of their increased percentage as free ion in solution. The left panel of Figure 6 displays the change in the extinction spectra concerning the formation of the metal derivative of porphyrin TPPS_4_ when titrated with Zn(II) ions. The spectroscopic features are in agreement with the findings in the literature, exhibiting a Soret band placed at 422 nm and two Q bands at 556 nm and 598 nm, respectively [65], although a certain extent of Rayleigh diffusion is also detectable. The corresponding fluorescence emission spectra acquired upon excitation at 418 nm are displayed in the right panel of Figure 6 and exhibit significant changes in the emission bands related to the free base porphyrin. In particular, a remarkable quenching of the fluorescence emission of the band positioned at lower energy is noticeable, whereas both a pronounced hypochromicity and contextual slight batochromic shift characterize the band at higher energy. On the other hand, a more relevant feature is the simultaneous formation of a new band centered at 422 nm, which is typically related to the formation of the Zn(II)TPPS_4_. The inset in the right panel of Figure 6 shows the trend of the fluorescence intensity measured at 645 nm when titrating the porphyrin with the Zn^2+^ ion in a concentration range of 0 to 280 µM, and from which a detection limit of 1.87 µM is calculated. Again, the eventual excitation at 380 nm will result in a moderate decrease in the fluorescence emission of GQDs ascribable to a secondary filter effect (Figure S4).

As mentioned above, in these changed experimental conditions, the cadmium ion is also detectable by the GQDs@TPPS_4_ system, as highlighted in the upper panel of Figure S5. The UV/vis spectra reveal the formation of the metal derivative featured by the occurrence of a new band located at 430 nm. The corresponding fluorescence emission spectra obtained by exciting at 423 nm are displayed in the lower panel of Figure S5 and show a progressive quenching of the fluorescence emission of the bands, whereas a detection limit of 0.60 µM can be calculated from the fluorescence intensity evolution recorded at 645 nm over a concentration range of 0 to 100 µM (inset lower panel of Figure S5).

**Figure 7:**
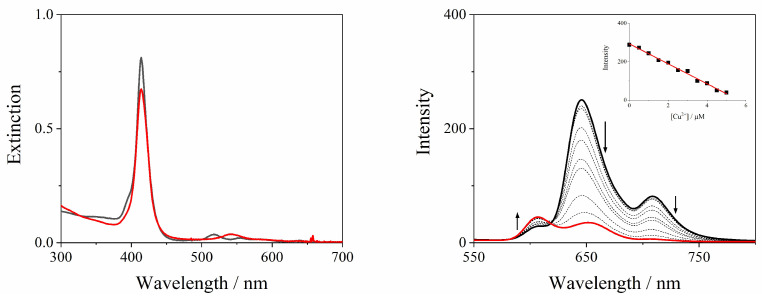
UV/Vis absorbance spectral changes (**left panel**) and corresponding fluorescence emission spectra variation exciting at λ = 418 nm (**right panel**) for GQDs@TPPS_4_ recorded, respectively, at the beginning (solid black line) and at the end (solid red line) of a titration with Cu^2+^ ions carried out at neutral pH (the arrow marks the increasing metal ion concentration). In the inset of the right panel are reported the corresponding fluorescence intensity changes at 645 nm, properly corrected for the occurrence of the zinc(II) derivative, and from which a LOD of 0.063 µM can be estimated (the red line represents the linear fit of the data). Experimental conditions: [TPPS_4_] = 3 μM; [GQDs] = 0.018 mg/mL; [Zn^2+^] = 30 μM; [Cu^2+^] = 0–5 μM; PBS 1 mM, pH = 7; T = 298 K; cell path length 1 cm.

Zinc is an essential trace element that plays a vital role in many physiological processes in the human body, but its adventitious presence in solutions may significantly impact both biological processes and chemical reactions, leading to a misinterpretation of findings. In this respect, in the recent past, this research group has already highlighted how aqueous solutions of TPPS_4_ under neutral pH conditions are able to strip out zinc(II) cations embedded in the surfaces of glass or silica, where it occurs as a factory additive [58]. However, other unexpected sources of metal contamination may be not only glassware, but also labware, including methacrylate cuvettes [56]. Such considerations prompted us to test the response of the system GQDs@TPPS_4_ to the metal ion presence by deliberately polluting all the employed solutions (including the solvent) with a known amount of zinc ion equal to 30 μM. This value is exactly the same as the one we have found in samples of commercial water for injectable solutions, and it is such that only a negligible percentage fraction of porphyrin is metalated as highlighted in Figure 6. Under these new experimental conditions, the responsiveness to the occurrence of copper ions remains quite effective. From the changes in UV/vis spectra reported in the left panel of Figure 7, it can be noted as the copper derivative of porphyrin is formed at fairly low concentrations of copper ions (5 μM). The corresponding fluorescence emission spectra reported in the right panel of Figure 7 show the quenching of fluorescence intensity of the bands related to TPPS_4_ and a concurrent slight increase in the intensity of the band positioned at 606 nm attributable to the zinc(II) derivative that was already present at the beginning due to the deliberate pollution of the solutions. In the inset is shown the trend of the fluorescence emission intensity recorded at 645 nm, which has been corrected for the occurrence of the zinc(II) derivative (see methods), and from which a LOD of 0.063 μM is computed, consistent with that already observed in Figure 2.

**Figure 8:**
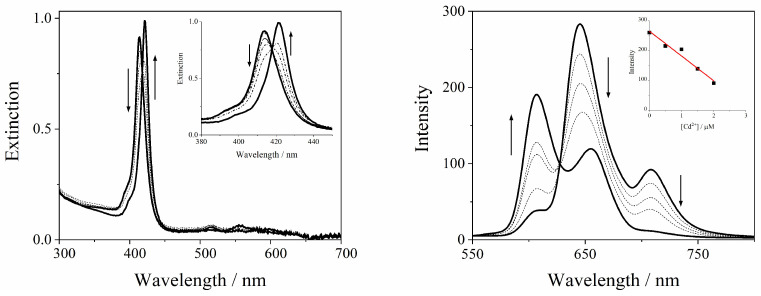
UV/Vis absorbance spectral changes (**left panel**) and corresponding fluorescence emission spectra variation exciting at λ = 418 nm (**right panel**) during the titration of GQDs@TPPS_4_ with Cd^2+^ ions carried out at neutral pH (the arrow marks the increasing metal ion concentration). In the inset of the right panel are reported the corresponding fluorescence intensity changes at 645 nm, properly corrected for the occurrence of the zinc(II) derivative, and from which a LOD of 0.039 µM can be estimated (the red line represents the linear fit of the data). Experimental conditions: [TPPS_4_] = 3 μM; [GQDs] = 0.018 mg/mL; [Zn^2+^] = 30 μM; [Cd^2+^] = 0–2 μM; PBS 1 mM, pH = 7; T = 298 K; cell path length 1 cm.

Unlike previous findings, in the presence of zinc(II) contamination, the system GQDs@TPPS_4_ appears to turn out to be responsive to cadmium(II) ions. In fact, upon addition of increasing amounts of these latter, UV/Vis analysis (Figure 8, left panel) reveals a gradual hypochromic shift of the B-band at 414 nm followed by the gradual appearance of a peak at 422 nm together with the formation of two Q-bands (540 and 590 nm) and the occurrence of an isosbestic point at 418 nm. Fluorescence emission spectra acquired through excitation at 418 nm (Figure 8, right panel) highlight a marked and progressive decrease in the emission intensity bands of the porphyrin together with a slight batochromic shift of the highest energy one (655 nm, Δλ = 10 nm) and a concurrent formation of a new blue-shifted band centered at 607 nm. The inset of the right panel of Figure 8 reports the progressive quenching of the emission fluorescence intensity at 645 nm when titrating with cadmium(II) ions. LoD of 0.039 μM can be calculated, a value which provides evidence of an enhanced sensitivity to cadmium(II) ions than that reported in Figure 4. All experimental findings, in agreement with that reported in Figure 6, would seem to support the formation in solution of the Zn(II)TPPS_4_. Similarly, a titration with mercury(II) ions will also lead to the formation of the zinc porphyrin (Figure S6), in contrast to what happens in the absence of metal ion pollution in solution.

Therefore, experimental results highlight that the adventitious presence of zinc(II) ions can be detected by the system TPPS_4_@GQDs, which acts as a fluorescent probe by specifically incorporating this metal ion through the assistance of large metal ions such as cadmium(II) and mercury(II). This finding is not surprising since it is known that both these non-environmentally friendly metal ions cannot fit properly into the porphyrin core but can instead be located on top of the porphyrin ring and distort its structure, leading to a “sitting-atop complex” (SAT), which promotes binding by smaller metal ions from the rear side [66]. Right in this regard and as previously mentioned, Qiu and coworkers have indeed reported that a SAT-like TMPyP porphyrin complex promoted by the mercury(II) ion is behind the mechanism by which the Mn(II) ion coordinated to NGQDs displaces the former metal in the porphyrin core [47]. Accordingly, in our specific case, the porphyrin metalation capability appears to be enhanced by the combination of two distinct catalytic strategies: ring deformation induced by a large radius ion promoting a SAT complex and the graphene adsorption effect.

### 2.2. GQDs@TPPS_4_ Metal Ion Coordination Efficiency

**Figure 9:**
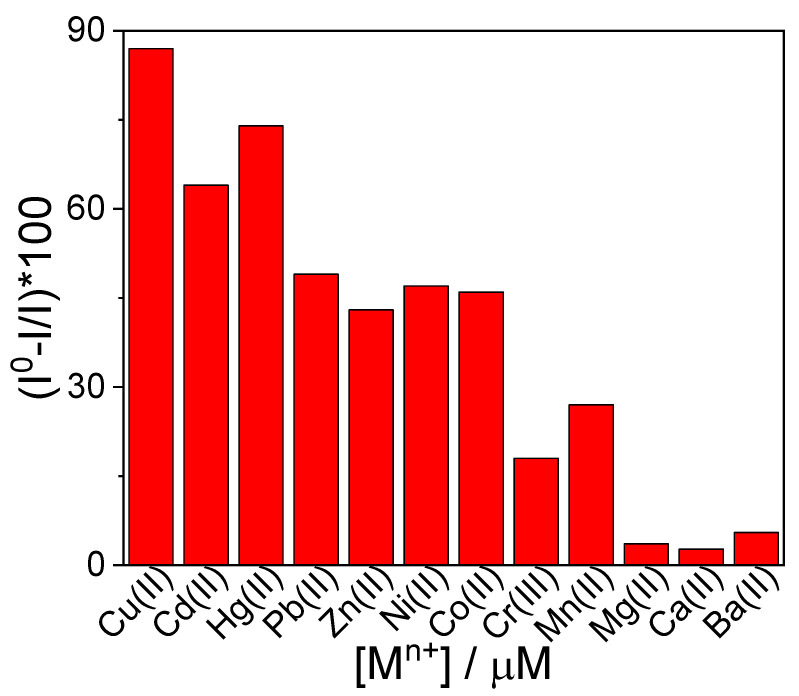
Fluorescence emission spectra showing quenching at a wavelength of 645 nm, excited at λ = 418 nm, of the GQDs@TPPS_4_ system induced by the interaction of several divalent cations. All data were properly corrected for the occurrence of the zinc(II) derivative. Experimental conditions: [TPPS_4_] = 3 μM; [GQDs] = 0.018 mg/mL; [Zn^2+^] = 30 μM; [M^2+^] = 4 μM; PBS 1 mM, pH = 7; T = 298 K; cell path length 1 cm.

To evaluate the selectivity of the proposed method, a series of experiments was carried out with a plethora of various metal cations, including some with environmental and biological relevance. The results obtained, keeping the concentration of each ion constant (4 μM), have shown a marked dependence of the fluorescence quenching efficiency on the nature of the metal cation, which followed the order (Figure 9):

Cu^2+^ > Hg^2+^ > Cd^2+^ > Pb^2+^ ~ Zn^2+^ ~ Co^2+^ ~ Ni^2+^ > Mn^2+^ ~ Cr^3+^ >> Mg^2+^ ~ Ca^2+^ ~ Ba^2+^

In general, due to the different structures determined by different metal ions, the stability and, thus, the ease of formation of metalloporphyrins is ranked in the following order [67,68]: medium metals > large metals > alkali metals, so here it is to be regarded as anomalous the position assumed by some cations. The less pronounced quenching effect of fluorescence emission is observed for larger and less polarizing cations as the alkaline-earth ones. This effect can be explained by a lower affinity for the porphyrin coordination site according to the so-called “Irving-Williams series” [69], which results in a smaller binding energy and, thus, a lower stability. Metal ions of medium radius (e.g., Cu^2+^, Zn^2+^) bind porphyrins in a 1:1 ratio (unlike alkali metals), coordinating all four nitrogen atoms and being perfectly allocated in the porphyrin ring [70,71]. All of this promotes the metal coordination reaction, prompting remarkable changes in terms of fluorescence emission of the dye, including, in some cases, its quenching. On the other hand, metal ions of even larger radius as Cd^2+^ and Hg^2+^ cannot fit into the porphyrin core, and they just sit on top of the porphyrin ring to form “sitting-atop” complexes [72,73,74]. In general, the porphyrin metalation rate is very slow due to the stable structure of the porphyrin ring and difficulty in its deformation. Here, the catalytic effect exhibited by these ions and, accordingly, their peculiar position in the sequence under investigation may be attributable to the further deformation effect determined by the formation of the SAT complex, which is additional to the presumably preexistent effect caused by the interaction of TPPS_4_ with GQDs.

## 3. Materials and Methods

### 3.1. Materials

Graphene Quantum Dots (GQDs) were prepared by microwave-assisted pyrolysis on a CEM Discover Synthesizer, Matthews, NC, U.S.A., by following a procedure recently reported in literature [48]. All chemicals were used without further purification and purchased in the highest available purity (≥99%) from commercial sources as follows: *meso*-tetrakis(4-sulfonatophenyl) porphyrin (TPPS_4_), as sodium salt (Frontier Scientific, Halstenbek, Germany); L-glutamic acid, triethylenetetramine tetrahydrochloride, sodium dihydrogenphosphate, and sodium hydrogen phosphate (Sigma-Aldrich, Milan, Italy); zinc sulfate hexahydrate, mercury(II) chloride, cadmium chloride, manganese(II) sulfate monohydrate, cobalt(II) chloride hexahydrate, magnesium chloride hexahydrate, calcium chloride dihydrate, barium sulfate, lead(II) nitrate, and nickel(II) chloride hexahydrate (VWR International, Milan, Italy). All the aqueous stock solutions of both porphyrin and various metal ions were prepared by dissolving the solids in high purity, doubly distilled water (HPLC grade, Fluka, Milan, Italy). Stock solutions of the porphyrins (~200 μM) were freshly prepared, kept in the dark to avoid photo-degradation and used within a week of preparation. The concentration of free TPPS_4_ porphyrin was determined by UV/Vis spectroscopy using the extinction coefficient of 5.33 × 10^5^ M^−1^ cm^−1^ and absorbance values at 414 nm [75].

### 3.2. Methods

UV-Visible extinction spectra were collected on an Agilent 8453 diode array UV-Vis spectrophotometer. The temperature was held constant at 298 K by an external water-circulating thermostatic bath. A Jasco FP-750 spectrofluorometer equipped with a Hamamatsu R928 phototube was used to collect the fluorescence emission spectra of the samples. The ζ-potential values were determined using a Zetasizer Nano ZS (Malvern Instrument, Malvern, UK) equipped with a 4 mW He-Ne laser (λ = 633 nm). pH measurements were obtained using an 827 pH Lab Metrohm meter (Formello, Rome, Italy) with a combined glass electrode (Metrohm 6.0228.010 with temperature sensor) calibrated with pH NIST buffers 4 and 7 (Merck CertiPUR^®^, Milan, Italy). Titration experiments have been carried out by recording UV/Vis absorbance and fluorescence emission spectra of solutions contained in 1 cm path length Hellma quartz cells and placed in the thermostatic holder of the instruments at a temperature of 298 K. The protocol adopted was to gradually add micromolar amounts of metal ions to a solution containing GQDs (0.018 mg/mL) and porphyrin (3 μM) at the experimental pH controlled by PBS 1/10 mM. All experimental data were collected right after the addition of the last reagent, at the solution equilibration, with no further time waiting. The LoD was calculated with the following equation: LoD = 3.3 SD/α, where SD is the standard deviation of blank measurement and α is the slope between the emission fluorescence intensity versus M^2+^ ion concentration. The kinetic data for the metalation of TPPS_4_ porphyrin followed a first-order law, and the estimated rate constant (*k_obs_*, s^−1^) was obtained from a nonlinear least square fit of the experimental extinction data at 540 nm to the Equation (1).

Ext_t_ = Ext_∞_ + (Ext_0_ − Ext_∞_) × exp(−*k_obs_* × *t*) (1)

where Ext_0_, Ext_∞_, and *k_obs_* are the parameters to be optimized (Ext_t_ = the extinction at time t; Ext_0_ = extinction soon after mixing reagents; Ext_∞_ = extinction at completion of the reaction). The secondary inner filter effect for the fluorescence emission of GQDs has been calculated by using the equation *I*_corr_ = *I*_obs_ × 10^Ext(λem)^, where *I*_corr_, *I*_obs_, and Ext(λ_em_) are the corrected fluorescence intensity, the observed fluorescence intensity, and the extinction of the sample at the emission wavelength. Where necessary, fluorescence emission intensity data were also corrected for the contribution related to the formation of the Zn(II)TPPS_4_ by using the following equation: I_645_ − (I_606_/1.71). I_645_ and I_606_ represent the fluorescence emission intensities at the particular wavelengths, whereas 1.71 is the experimental value of the ratio between the fluorescence emission intensities of the bands related to the porphyrin Zn(II)TPPS_4_ interacting with GQDs in the same experimental conditions adopted in the main text. Equilibrium constants at *I* ~ 0.03 M and *T* = 298.15 K used to draw distribution diagrams with PyES software are taken from NIST46 database [76]

## 4. Conclusions

The supramolecular adduct GQDs@TPPS_4_ can be designed to exhibit fluorescence emission from both the constituent fluorophores, i.e., graphene QDs and the porphyrin. In line of principle, this feature could lead to the development of a ratiometric sensor for metal ions. Actually, the nanosystem in our hands is able to detect the presence of a series of relevant species, even if a partial quenching of the GQD emission makes it a difficult ratiometric application without the post-processing of the data to eliminate filter effects. In any case, the rate of metal insertion into the porphyrin macrocycle is enhanced by the presence of GQD_S,_ and it is specifically accelerated by large metal ions, i.e., Hg^2+^ and Cd^2+,^ that form sitting-atop complexes able to catalyze the incorporation of adventitious Zn^2+^ ions. This latter behavior could lead to the potential development of a sensitive sensor based on the formation of Zn(II)TPPS_4,_ able to detect some metal ions in a submicromolar range of concentration.

## Figures and Tables

**Table 1:** Percentage (±5%) of uncomplexed divalent cations (c_M_ = 30 μM) in aqueous phosphate buffer ([PO_4_]_T_ = 1 and 10 mM, pH = 7) at T = 298.15 K.

Metal Cation	[PO_4_]_T_ = 1 mM	[PO_4_]_T_ = 10 mM
Zn^2+^	53%	10%
Cd^2+^	67%	17%
Cu^2+^	50% ^a^	9.5% ^b^
Hg^2+^	98.5% ^c^	89% ^d^

^a^ as sum of 42% free Cu^2+^ and 8% of Cu(OH)^+ b^ as sum of 8% free Cu^2+^ and 1.5% of Cu(OH)^+ c^ as sum of 92.5% Hg(OH)_2_(aq) and 6% of Hg(OH)Cl(aq) ^d^ as sum of 84% Hg(OH)_2_(aq) and 5% of Hg(OH)Cl(aq).

## Data Availability

Data are contained within the article.

## Supplementary Materials

The following supporting information can be downloaded at: https://www.mdpi.com/article/10.3390/ijms26157295/s1.
