# Supplementary material for: Supramolecular Graphene Quantum Dots/Porphyrin Complex as Fluorescence Probe for Metal Ion Sensing
Source: Int J Mol Sci. 2025 Jul 28;26(15):7295. doi: 10.3390/ijms26157295 (PMC12347067; doi:10.3390/ijms26157295)
Supplement: Supplementary file 1 [file ijms-26-07295-s001.zip › ijms-3773755-supplementary.pdf]

Supporting information for

Supramolecular Graphene Quantum Dots/Porphyrin Complex as Fluorescence Probe for Metal Ion Sensing

Mariachiara Sarà, Andrea Romeo, Gabriele Lando, Maria Angela Castriciano, Roberto Zagami, Giovanni Neri and Luigi Monsù Scolaro

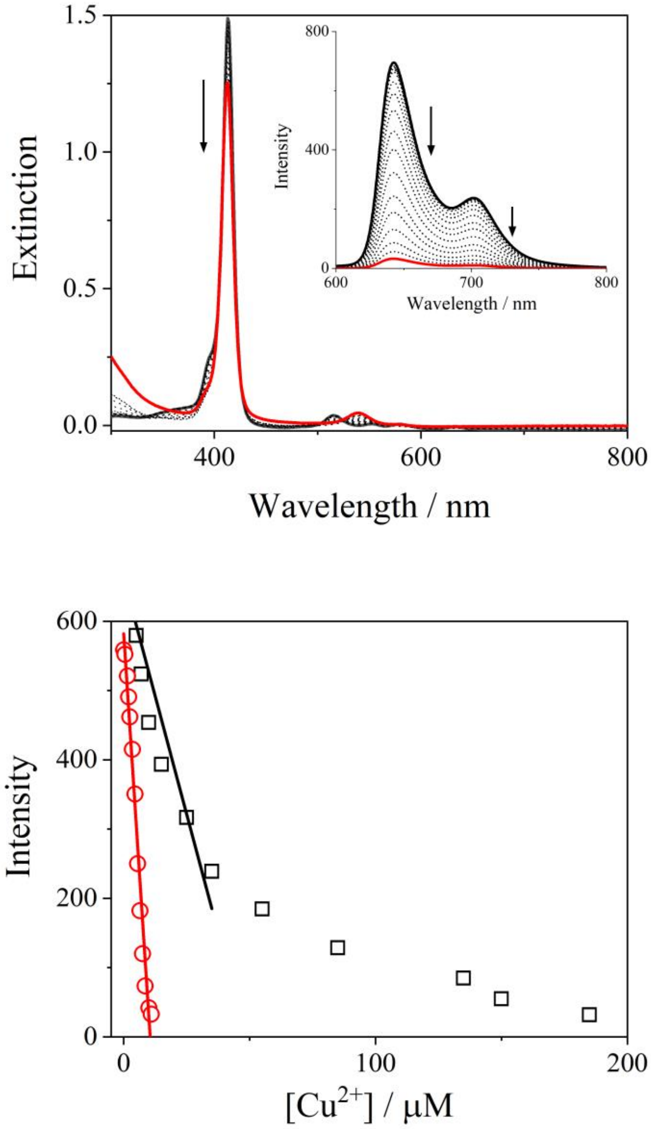

Figure S1. UV/Vis absorbance spectral changes (**upper panel**) and corresponding fluorescence emission spectra variation exciting at λ = 420 nm (**upper panel inset**) for the titration of $TPPS_4$ with Cu^{2+} ions carried out at neutral pH in bulk (the arrow marks the increasing metal ion concentration). In the **lower panel** (solid black line) are reported the corresponding fluorescence intensity changes at 645 nm from which a LoD = 0.24μM is calculated. As a comparison, in the same inset with a red line is reported the result achieved by interaction with GQDs as described in the main text (LoD = 0.059μM). Experimental conditions: [$TPPS_4$] = 3 μM; [Cu^{2+}] = 0-200 μM; PBS 10 mM, pH = 7; T= 298 K; cell path length 1 cm.

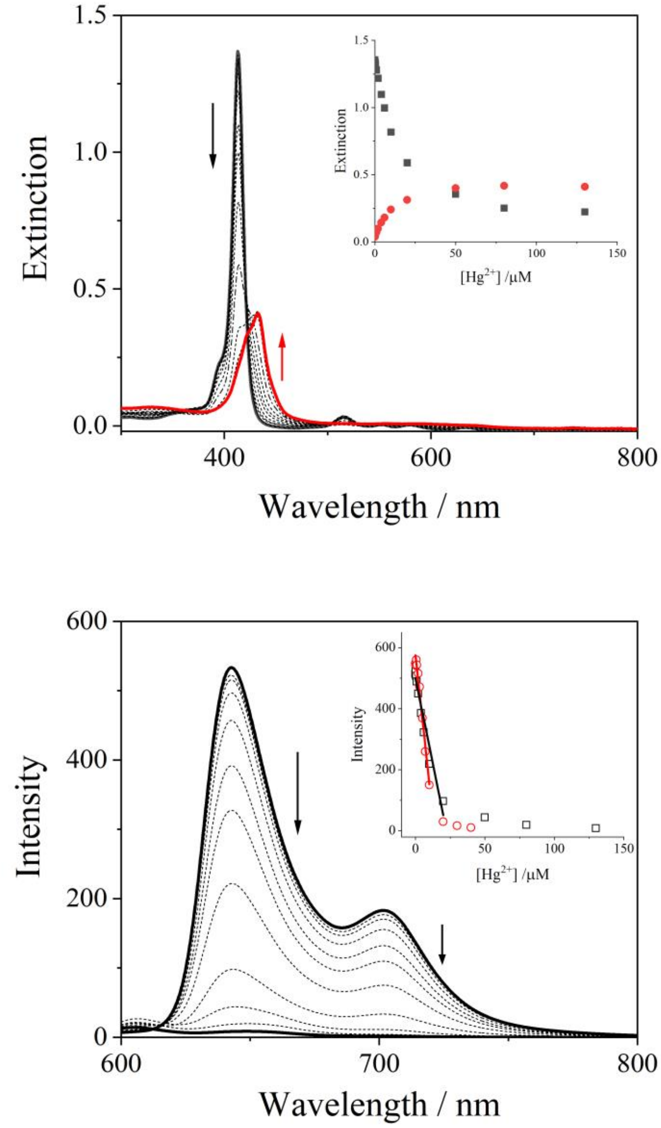

Figure S2. UV/Vis absorbance spectral changes (**upper panel**) and corresponding fluorescence emission spectra variation exciting at λ = 422 nm (**lower panel**) for the titration of $TPPS_4$ with Hg^{2+} ions carried out at neutral pH in bulk (the arrow marks the increasing metal ion concentration). In the **upper panel inset** are shown the extinction time trends corresponding to 414 nm (black squares) and 432 nm (red circles) while in the **lower panel inset** are reported the corresponding fluorescence intensity changes at 645 nm (black squares) from which a LoD = 0.15μM is calculated. As a comparison, in the same inset with a red line is reported the result achieved by interaction with GQD_S as described in the main text (LoD = 0.078μM). Experimental conditions: [$TPPS_4$] = 3 μM; [Hg^{2+}] = 0-150 μM; PBS 10 mM, pH = 7; T= 298 K; cell path length 1 cm.

Equilibrium	log *K*
$H_2O = H^+ + OH^-$	-13.8
$H^+ + PO_4^{3-} = HPO_4^{2-}$	11.49
$2\ H^+ + PO_4^{3-} = H_2PO_4^-$	18.14
$3\ H^+ + PO_4^{3-} = H_3PO_4^0{}_{(aq)}$	20.01
$Zn^{2+} + H_2O = Zn(OH)^+ + H^+$	-9.22
$Zn^{2+} + 2\ H_2O = Zn(OH)_2^0{}_{(aq)} + 2\ H^+$	-17.14
$Zn^{2+} + 3\ H_2O = Zn(OH)_3^- + 3\ H^+$	-28.46
$Zn^{2+} + 4\ H_2O = Zn(OH)_4^{2-} + 4\ H^+$	-40.68
$Cd^{2+} + H_2O = Cd(OH)^+ + H^+$	-9.8
$Cd^{2+} + 2\ H_2O = Cd(OH)_2^0{}_{(aq)} + 2\ H^+$	-20.2
$Cd^{2+} + 3\ H_2O = Cd(OH)_3^- + 3\ H^+$	-33.5
$Cd^{2+} + 4\ H_2O = Cd(OH)_4^{2-} + 4\ H^+$	-47.28
$Cu^{2+} + H_2O = Cu(OH)^+ + H^+$	-7.7
$Cu^{2+} + 2\ H_2O = Cu(OH)_2^0{}_{(aq)} + 2\ H^+$	-15.8
$Cu^{2+} + 3\ H_2O = Cu(OH)_3^- + 3\ H^+$	-26.9
$Cu^{2+} + 4\ H_2O = Cu(OH)_4^{2-} + 4\ H^+$	-39.6
$Hg^{2+} + Cl^- = HgCl^+$	6.7
$Hg^{2+} + 2\ Cl^- = HgCl_{2(aq)}$	13.5
$Hg^{2+} + 3\ Cl^- = HgCl_3^-$	14.5
$Hg^{2+} + 4\ Cl^- = HgCl_4^{2-}$	15.2
$Hg^{2+} + H_2O = Hg(OH)^+ + H^+$	-3.2
$Hg^{2+} + 2\ H_2O = Hg(OH)_2^0{}_{(aq)} + 2\ H^+$	-6.4
$Hg^{2+} + Cl^- + H_2O = HgCl(OH)^0{}_{(aq)} + H^+$	3.65
$Hg(OH)_2^0{}_{(aq)} + 2\ H^+ = Hg^{2+} + 2\ H_2O$	2.16
$Zn^{2+} + PO_4^{3-} = ZnPO4^-$	7.3
$Zn^{2+} + H^+ + PO_4^{3-} = ZnHPO_4^0{}_{(aq)}$	14.3
$Cd^{2+} + H^+ + PO_4^{3-} = CdHPO_4^0{}_{(aq)}$	14.34
$Cd^{2+} + 2\ H^+ + PO_4^{3-} = CdH_2PO_4^-$	18.84
$Cu^{2+} + H^+ + PO_4^{3-} = CuHPO_4^0{}_{(aq)}$	14.69
$Cu^{2+} + 2\ H^+ + PO_4^{3-} = CuH_2PO_4^-$	18.78
$Hg^{2+} + PO_4^{3-} = HgPO_4^-$	9.5
$Hg^{2+} + H^+ + PO_4^{3-} = HgHPO_4^0{}_{(aq)}$	20.29
$Hg^{2+} + H^+ + PO_4^{3-} = HgHPO_4^0{}_{(s)}$	-1.61

Table S1. Equilibrium constants at I ~ 0.03 M and T = 298.15 K used to draw distribution diagrams of the various studied systems taken from NIST46 database [1]. When data at I = 0.03 were not available they were estimated using well known extended Debye-Hückel type equations. The stability constants of $ZnCl^+$, $CuCl^+$ and $CdCl^+$ species as well as all the polynuclear hydrolytic species were neglected for simplicity.

$[Zn]_T = 30\ \mu M;\ [PO_4]_T = 1\ mM$

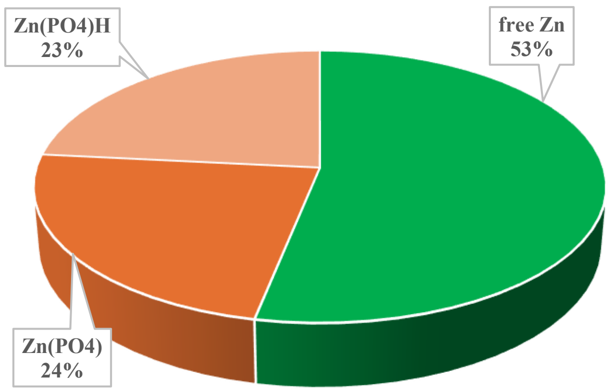

$[Zn]_T = 30\ \mu M;\ [PO_4]_T = 10\ mM$

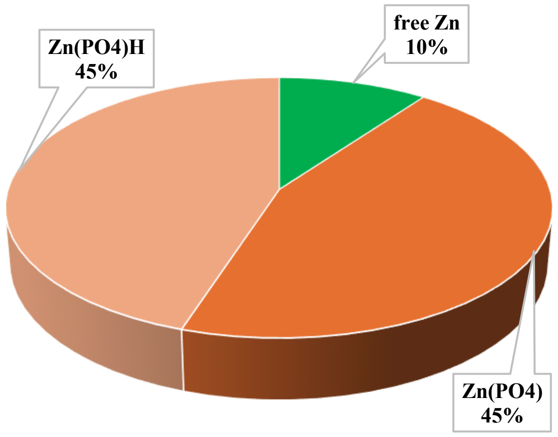

$[Cd]_T = 30\ \mu M;\ [PO_4]_T = 1\ mM$

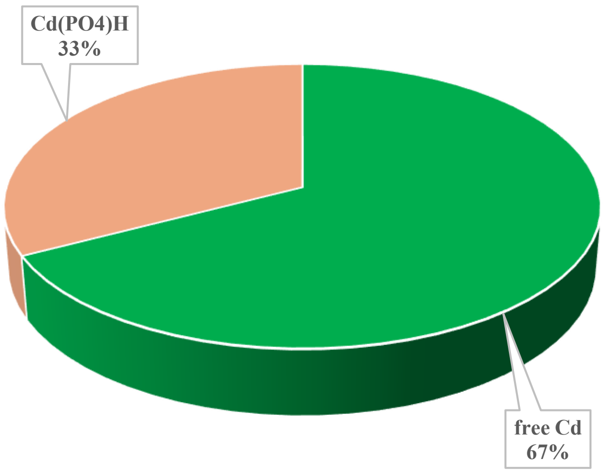

$[Cd]_T = 30\ \mu M;\ [PO_4]_T = 10\ mM$

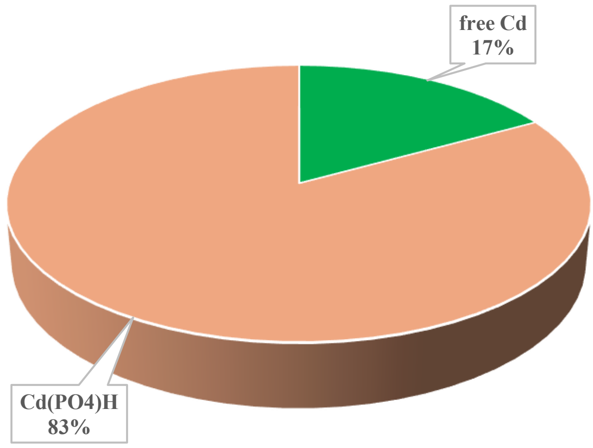

$[Cu]_T = 30\ \mu M;\ [PO_4]_T = 1\ mM$

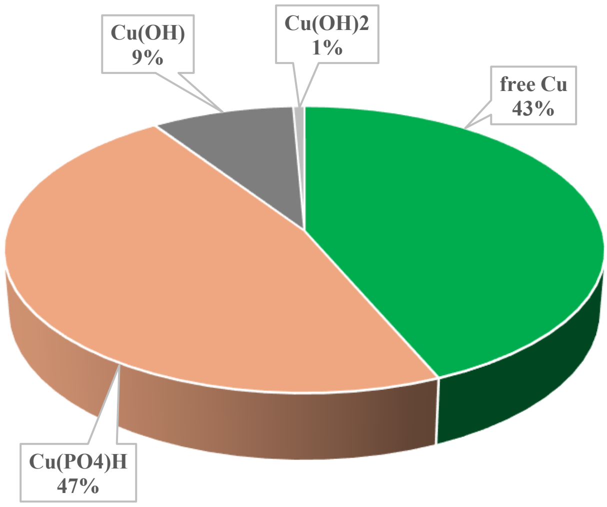

$[Cu]_T = 30\ \mu M;\ [PO_4]_T = 10\ mM$

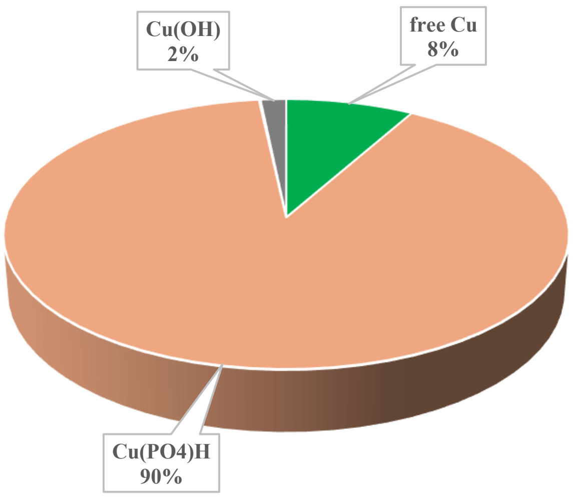

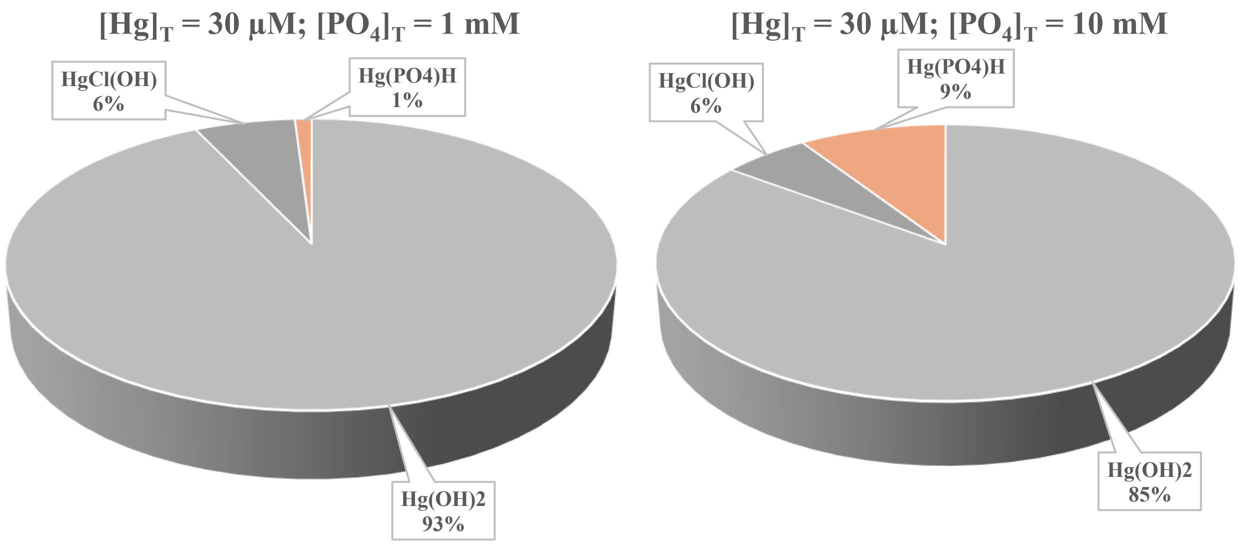

Figure S3. Pie chart diagrams reporting the distribution of the species of the four $M^{2+}/PO_4^{3-}/H^+$ systems at pH = 7 drawn with PyES software [2]. Species with a formation percentage below 1 % were ignored. Color code: green = free hydrated cation; orange scale = variously protonated phosphate complex species; grey scale = simple or mixed hydrolytic species.

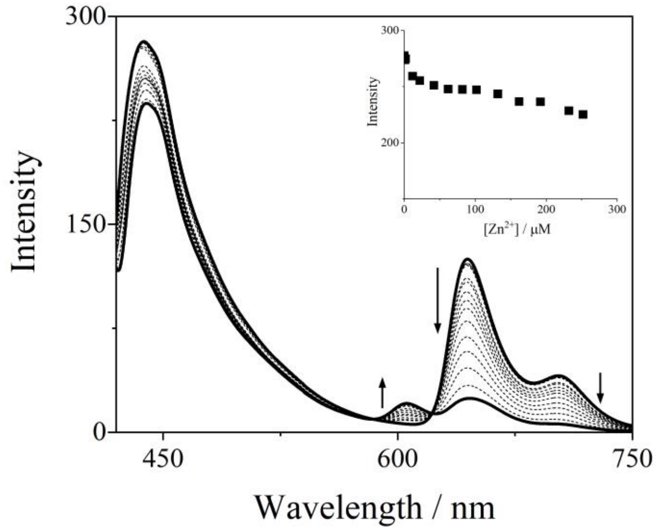

Figure S4. Fluorescence emission spectra variation exciting at λ = 380 nm for the titration of GQDs/$TPPS_4$ with Zn^{2+} ions carried out at neutral pH (the arrow marks the increasing metal ion concentration). In the **inset** are shown the fluorescence emission trend at 435 nm properly corrected for the filter effect induced by the absorption of the porphyrin metal derivative. Experimental conditions: [$TPPS_4$] = 3 μM; [GQDs] = 0.018 mg/mL; [Zn^{2+}] = 0-280 μM; PBS 1 mM, pH = 7; T= 298 K; cell path length 1 cm.

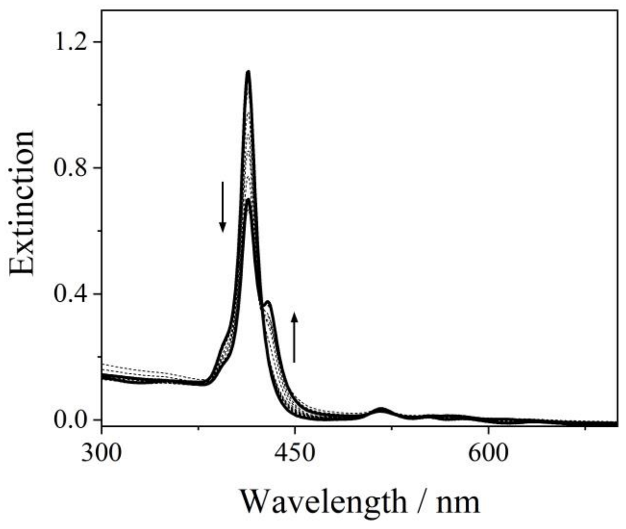

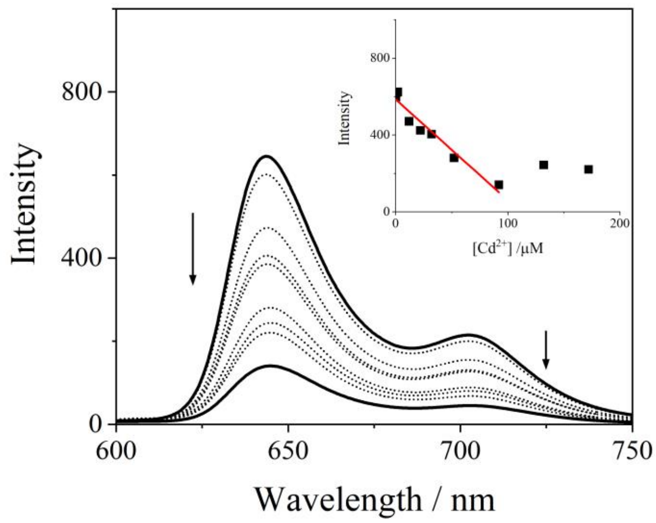

Figure S5. UV/Vis absorbance spectral changes (**upper panel**) and corresponding fluorescence emission spectra variation exciting at λ = 423 nm (**lower panel**) for the titration of GQDs/$TPPS_4$ with Cd^{2+} ions carried out at neutral pH (the arrow marks the increasing metal ion concentration). In the **lower panel inset** are reported the corresponding fluorescence intensity changes at 645 nm from which a LoD = 0.60μM is calculated. Experimental conditions: [$TPPS_4$] = 3 μM; [GQDs] = 0.018 mg/mL; [Cd^{2+}] = 0-180 μM; PBS 1 mM, pH = 7; T= 298 K; cell path length 1 cm.

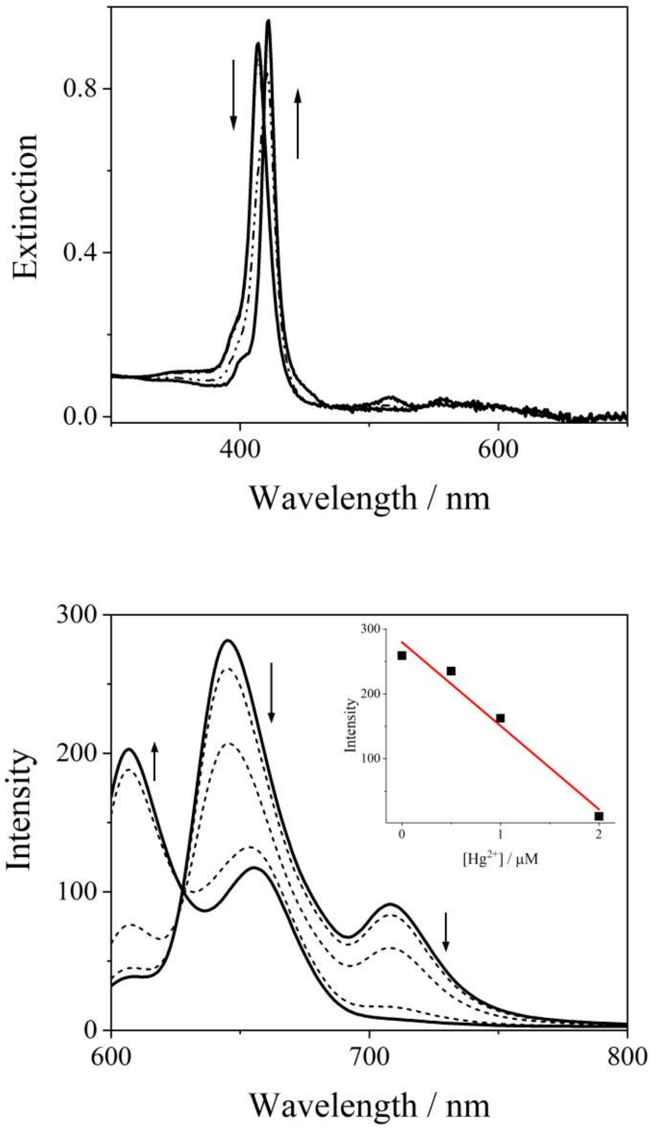

Figure S6. UV/Vis absorbance spectral changes (**upper panel**) and corresponding fluorescence emission spectra variation exciting at λ = 418 nm (**lower panel**) for the titration of GQDs/$TPPS_4$ with Hg^{2+} ions carried out at neutral pH (the arrow marks the increasing metal ion concentration). In the **lower panel inset** are reported the corresponding fluorescence intensity changes at 645 nm properly corrected for the zinc(II) derivative and from which a LoD = 0.025μM is calculated. Experimental conditions: [$TPPS_4$] = 3 μM; [GQDs] = 0.018 mg/mL; [Zn^{2+}] = 30 μM; [Hg^{2+}] = 0-12 μM; PBS 1 mM, pH = 7; T= 298 K; cell path length 1 cm.

References

[1] Martell, A.; Smith, R.; Motekaitis, R., NIST standard reference database 46, vers. 8. US Department of Commerce, Gaithersburg, Md, USA **2004**.

[2] Castellino, L.; Alladio, E.; Bertinetti, S.; Lando, G.; De Stefano, C.; Blasco, S.; García-España, E.; Gama, S.; Berto, S.; Milea, D., PyES – An open-source software for the computation of solution and precipitation equilibria. *Chemom. Intell. Lab. Syst.* **2023,** 239, 104860.
